# Usual Choline Intakes Are Associated with Egg and Protein Food Consumption in the United States

**DOI:** 10.3390/nu9080839

**Published:** 2017-08-05

**Authors:** Taylor C. Wallace, Victor L. Fulgoni

**Affiliations:** 1 Department of Nutrition and Food Studies, George Mason University, 10340 Democracy Lane, Suite 306, Fairfax, VA 22030, USA; 2 Think Healthy Group, Inc., 127 U Street NW, Washington, DC 20001, USA; 3 Nutrition Impact, LLC, 9725 D Drive North, Battle Greek, MI 49014, USA; vic3rd@aol.com

**Keywords:** choline, NHANES, usual intake, adequate intake, tolerable upper intake level, dietary reference intake

## Abstract

Choline is an essential nutrient with critical roles in several biological processes including neuronal development, cell signaling, nerve impulse transmission, and lipid transport and metabolism. The National Cancer Institute method was used to assess usual intakes of choline from foods according to data for participants enrolled in the National Health and Nutrition Examination Survey 2009–2014 datasets and pregnant women in the 2005–2014 datasets. Suboptimal intakes of choline are present across many gender and life-stage subpopulations, as well as pregnant women in the U.S. Only 8.03 ± 0.56% of adults and 8.51 ± 2.89% pregnant women meet the AI for choline. Children 2–3 years were the most likely to meet their gender and life-stage specific AI, followed by children 4–8 years. Adults 19+ years who consume eggs were more likely to meet their gender and life-stage AI as compared to non-consumers (57.3 ± 1.45% and 2.43 ± 0.28%). Consumers of eggs had almost double the usual intake of choline as compared to non-consumers (525 ± 5.17 mg/d and 294 ± 1.98; *p* < 0.0001). Protein food (meat, poultry and seafood) consumption also increased usual choline intakes compared to non-consumers (345 ± 2.21 mg/day and 235 ± 8.81; *p* < 0.0001) to a lesser degree, but did not result in substantial increases in the percent of individuals meeting the AI. No subpopulation exceeded the UL for choline. This research illustrates that it is extremely difficult to achieve the AI for choline without consuming eggs or taking a dietary supplement.

## 1. Introduction

Choline is an essential micronutrient with critical roles in several biological processes [1]. As a source of methyl groups, choline supports cellular methylation reactions, including genomic methylation, which impacts gene expression, the cell membrane that signal the stability of DNA and lipid transport and metabolism. It also serves as the substrate for the formation of acetylcholine, a neurotransmitter and non-neuronal cell-signaling molecule that is important for memory, mood, muscle control, and other brain and nervous system functions. The metabolic fate of choline is the biosynthesis of phosphatidylcholine and sphingomyelin, which are major phospholipids within cell membranes that are critical for structural integrity [1,2,3]. Humans can produce small amounts of choline endogenously in the liver, primarily in the form of phosphatidylcholine; however, the amount is not sufficient to meet human requirements for the micronutrient [4]. Therefore, humans must obtain choline from the diet. The requirement for choline may be different among subpopulations and rises when the diet is deficient in folate, since it becomes the primary methyl donor [2]. Choline is vital for early brain development [1,2], which might be why estrogen in premenopausal women induces the gene that catalyzes natural biosynthesis of choline [4]. Choline deficiency results in non-alcoholic fatty liver disease, because it is required to form the phosphatidylcholine present in very low-density lipoprotein (VLDL) particles [1]. Deficiency can also lead to neural tube defects during pregnancy and suboptimal brain development among the fetus and in infants [5].

The most common sources of choline in foods are fat-soluble phosphatidylcholine and sphingomyelin as well as water-soluble phosphocholine, glycerolphosphocholine, and free choline [2]. Pancreatic and mucosal enzymes liberate free choline from about half of the fat-soluble forms and some water-soluble forms [6]. Foods naturally containing choline include chicken liver (3 oz.; 247 mg), salmon (3 oz.; 187 mg), eggs (1 large egg with yolk; 125 mg), shitake mushrooms (1/2 c.; 58 mg), chicken broilers or fryers (3 oz.; 56 mg), beef grass-fed strip steak (3 oz.; 55 mg), wheat germ (1 oz. toasted; 51 mg), milk (8 oz.; 38 mg), brussels sprouts (1/2 c.; 32 mg), and almonds (1 oz.; 15 mg). Eggs represent the major source of choline in the US diet [7]. In 1998, the US National Academies of Medicine (NAM) Food and Nutrition Board (FNB) established an adequate intake (AI) and a tolerable upper intake level (UL) for choline. At the time, the FNB felt that the existing peer-reviewed literature was insufficient to determine an estimated average requirement (EAR) or recommended dietary allowance (RDA) for choline, so an AI was set based on the prevention of non-alcoholic fatty liver disease in two studies of adult men [1]. The AI for infants reflects the observed mean intake from human breast milk. The UL was derived from the lowest observed adverse effect level shown to cause hypotension in adults (i.e., 3.50 g/day) [1]. Dietary reference intakes for choline are illustrated in Table 1.

When the AI for choline was established in 1998, it was not known whether there were significant numbers of individuals who were choline deficient, and until recently, little data from food composition databases have been available on the choline content of foods [1]. Our previous work found large portions (approximately 90%) of the US population age ≥2 years to have suboptimal intakes of choline [8]. Thus, this manuscript seeks to explore choline intakes among consumers vs. non-consumers of eggs, protein foods (meat, poultry and seafood), and dietary supplements.

## 2. Materials and Methods 

### 2.1. Study Population

The National Center for Health Statistics (NCHS) of the U.S. Centers for Disease Control and Prevention administers and collects the National Health and Nutrition Examination Survey (NHANES), a nationally representative, cross-sectional survey of noninstitutionalized, civilian U.S. residents [9]. The NHANES survey protocol was approved by the Research Ethics Review Board of the NCHS. Written informed consent was obtained for all survey participants. Data from NHANES 2009–2010, 2011–2012, and 2013–2014 were combined for these analyses, except for pregnant women, which combined data from NHANES 2005–2006, 2007–2008, 2009–2010, 2011–2012, and 2013–2014. The combined sample included 24,774 participants and 593 pregnant women who had completed and provided 24-hour dietary intake data. In addition to dietary intake data, participants were asked to provide other information inclusive of, but not limited to, their gender, age, race, weight, household income level, and vegetarian status. Subjects who were aged <2 years were excluded from these analyses. 

### 2.2. Dietary Choline

NHANES participants were asked to complete two dietary recall interviews, with the first collected in person by trained interviewers. Proxy respondents provided dietary information for young children and proxy-assisted interviews were used for children aged 6–11 years. The second dietary recall interview was completed by telephone 3–10 days after the health examination. The U.S. Department of Agriculture’s (USDA) Automated Multiple-Pass Method was utilized for both dietary recall interviews [10,11]. Questionnaires, data sets, and related documentation from each NHANES analysis can be found on the NCHS NHANES website [12]. Various USDA food composition databases were utilized to determine the micronutrient contribution of specific foods consumed by NHANES participants. The USDA estimated the choline content of foods in recipes by linking the ingredients in survey food recipes to food composition data provided by the USDA National Nutrient Database for Standard Reference [13].

### 2.3. Supplemental Choline

Information on the use of dietary supplements (including vitamins, minerals, herbs, and so forth) over the previous 30 days prior to the dietary recall interview was collected as part of the dietary supplement questionnaire. Detailed information was obtained for each reported dietary supplement, including the frequency of consumption (i.e., the number of days the product was taken in the past 30 days), duration of use (i.e., how many days, weeks, months, or years the product was taken), and the amount normally taken per day on days it was taken over the 30-day period. The interviewer also examined each dietary supplement container and recorded complete product information so that each dietary supplement could be matched or entered into a database. The average daily intake of choline obtained from dietary supplements was calculated by using the number of days use was reported, the reported amount taken daily, and the serving size unit from the Supplement Facts Panel. 

### 2.4. Definitions

Gender was defined as male or female. DRI age groups (2–3, 4–8, 9–13, 14–18, 19–30, 31–50, 51–70, and ≥71 years) were used to compare estimates of choline intakes. Children and adults were defined as those individuals who were aged between 2 and 18 years and ≥19 years, unless otherwise specified. 

### 2.5. Comparison to DRI Values

The DRIs are a family of nutrient reference values, defined by the NAM Food and Nutrition Board, intended to serve as a guide for good nutrition and to provide the basis for the development of nutrient guidelines in both the United States and Canada. In 1998, the NAM updated and established an AI, and UL for choline [1]. These intake recommendations are specific to the role of choline in preventing non-alcoholic fatty liver disease. The values differ for individuals based on age and gender. Choline was reported as usual intake, as well as the percentage of the population with usual intakes above the AI and UL. 

### 2.6. Statistical Analysis

The National Cancer Institute (NCI) method as previously described [14] was used to determine estimates of usual choline intakes from the diet. The covariates used in the NCI model were as follows: (1) sequence of 24-hour recall, (2) day of the week the 24-hour recall was collected, and (3) dietary supplement use. All statistical analyses were performed with SAS software (version 9.2; SAS Institute Inc., Cary, NC, USA). SAS macros necessary to fit this model and to perform the estimation of usual intake distributions as well as additional details and resources regarding the NCI method are available on the NCI website [15]. Sample weights were used to account for differential response and non-coverage and to adjust for planned over-lapping of some groups. Survey weights were also used to generate a nationally representative sample. Mean dietary intakes of choline between users and nonusers of eggs and/or protein foods (meat, poultry and seafood) and the portion that met the AI and exceeded the UL were compared by computing a z statistic. Significance was set at *p* value of <0.01. 

## 3. Results

This manuscript expands our previous work (8) with more current population data, as well as data in pregnant women, toddlers, and consumers vs. non-consumers of choline-rich eggs and protein foods (meat, poultry and seafood). Only 11.3 ± 0.47% of the U.S. population ≥2 years met the AI for choline. Among the general adult population age 19+ y in the U.S., only 8.03 ± 0.56% meet the AI for choline (Table 2). Adult males were more likely than adult females to meet their age and gender specific AI (12.7 ± 0.95% and 3.61 ± 0.58%; *p* ≤ 0.0001). Adults 19+ y had a usual intake of 338 ± 1.86 mg/d (males: 405 ± 3.30 mg/d; females: 273 ± 2.13 mg/d). Usual intakes of choline increased with age until age 51–70 years. Pregnant women had a usual intake of 319 ± 9.89 mg/d and only 8.51 ± 2.89% of pregnant women 13–44 years met the AI for choline. Children 2–3 years were the most likely to meet their gender and life-stage specific AI, followed by children 4–8 years. Older children 9 + years and adults had a low prevalence of meeting the AI for choline. Teenagers 14–18 years were the most likely subpopulation to not meet the AI for choline. Children age 2-18 years had a usual intake of 252 ± 2.21 mg/d (males: 276 ± 2.96; females: 228 ± 2.51). 

Egg consumption was associated with higher usual intakes of choline across all subpopulations (*p* < 0.0001). Adults 19+ years who consume eggs were more likely to meet their gender and life-stage AI as compared to non-consumers (57.3 ± 1.45% and 2.43 ± 0.28%) (Table 3). Adults that consumed eggs on day one of NHANES obtained almost double the usual intake of choline as compared to non-consumers (525 ± 5.17 mg/d and 294 ± 1.98; *p* ≤ 0.0001). The relationship between egg consumption Healthy Eating Index 2010 (HEI-2010) scores was small and varied across subpopulations. Adults who consumed eggs were more likely to have a slightly higher HEI-2010 score as compared to non-consumers (51.7 ± 0.40 vs. 50.3 ± 0.27; *p* = 0.0037), however these differences were not statistically significant among adults age 31–50 or 51–70 years (data not shown). We did not identify any significant differences in the percent of egg consumers vs. non-consumers in regard to gender, ethnicity, weight status, smoking status, or amount of physical activity that were consistent across age and life-stage subpopulations (data not shown). The substitution of 2 eggs per day for red and/or processed meat resulted in a Healthy US-style 2000-kcal food pattern model that enables the AI for choline to be met (Table 4).

Protein food (meat, poultry and seafood) consumption was also associated with also significantly associated with higher usual intakes of choline across all subpopulations. Usual choline intakes increased in consumers vs. non-consumers (345 ± 2.21 mg/d and 235 ± 8.81; *p* ≤ 0.0001), but to a lesser degree as compared to egg consumers vs. non-consumers (Table 5). A small but significant increase in the percent of individuals who met the AI was noted in protein food consumers vs. non-consumers (8.62 ± 0.64% and 2.32 ± 0.89%; *p* = 0.0000). The relationship between protein food consumption and Healthy Eating Index 2010 (HEI-2010) scores was also small and varied across subpopulations. Adults who consumed protein foods were more likely to have a slightly lower HEI-2010 score as compared to non-consumers (59.2 ± 1.24 vs. 50.6 ± 0.26; *p* ≤ 0.0001), however these differences were not statistically significant among adults age 71+ years, males 19–30, 51–70, and 71+ years, or females 31–50 or 71+ years (data not shown). We did not identify any significant differences in the percent of protein food consumers vs. non-consumers in regard to gender, ethnicity, weight status, smoking status, or amount of physical activity that were consistent across age and life-stage subpopulations (data not shown).

Usual choline intakes from dietary supplements are consistent with the amounts reported in our previous work on multivitamins [8]. Multivitamins and other dietary multi-nutrient supplements contain only minimal amounts of choline, since choline salts are bulky and vastly increase the size of supplement product. Only minuscule portions of the U.S. population, including pregnant women, consume choline as a single-nutrient supplement, making it impossible to assess their impact on usual choline intakes from a population level. Dietary supplements provided approximately 13.83 ± 0.97 mg/day of choline to the diets of children 2–18 years and 25.2 ± 2.72 mg/day to that of adults 19+ years. Consistent use of dietary supplements did slightly but significantly increase usual choline intakes across all subpopulations (*p* < 0.0001) (Table 6).

We did not identify any population where intakes of choline exceeded (or came close to exceeding) the UL, even when supplements were included. At the 95th percentile males 31–50 years had the highest usual intakes of choline (652 ± 11.6 mg/day) which is well under the 3500 mg/day UL for this subpopulation.

## 4. Discussion

There is a clear need to increase awareness among health professionals, policy makers, and consumers regarding the large portion of individuals and subpopulations who do not currently achieve the AI for choline in the United States. It is necessary for the National Academy of Medicine, Food and Nutrition Board to update Dietary Reference Intakes (DRIs) for choline so that we may more accurately assess the magnitude of concern about potential population inadequacies for any nutrient with an assigned AI. Lack of an Estimated Average Requirement (EAR) severely limits the interpretation of the population intake data because it is difficult to assess whether intake below the AI results in suboptimal health status (e.g., impaired liver function, cognition, etc.). Furthermore, the USDA food composition databases are somewhat limited in their estimations of the choline content of foods. The databases do not currently accurately account for many branded food products. Despite these limitations, our data and that previously published online by our group [8], the USDA [16], and other international populations in Europe [17], Taiwan [18], New Zealand [19], and Canada [20] consistently illustrate that intakes of choline fall well below the AI for many subpopulations (particularly pregnant and/or lactating women), and that may pose a significant public health concern. We did not identify any significant differences in the percent of protein food consumers vs. non-consumers in regard to gender, ethnicity, weight status, smoking status, or amount of physical activity.

The 2015–2020 Dietary Guidelines for Americans Advisory Committee (DGAC) determined that of the nutrients with an AI (vitamin K, choline, dietary fiber, and potassium), that a low proportion of the population had fiber and potassium intakes above the AI, and therefore considered them to be under-consumed [21]. These data strongly suggest that choline is also under-consumed by the U.S. population, to a similar extent. The 2015–2020 DGAC also found that adequacy goals were not met in almost all food patterns for potassium, vitamin D, vitamin E and choline [17]. This is surprising given that the recent DGAC eliminated the long-standing recommendations around total cholesterol, which enables eggs to be included in food pattern models to a greater extent. Our food pattern models show that substituting red and/or processed meat with two eggs per day enables consumers to meet the AI for choline without compromising other nutrient intakes. When young healthy adults consumed 1, 2, or 3 eggs/day for 4-week serum trimethylamine-N-oxide (TMAO), a byproduct of choline which has been thought to increase the risk of heart disease, levels did not increase [22]. Egg consumption in this study did increase HDL-cholesterol but not LDL-cholesterol [22], thus reaffirming their utility to safely increase choline intakes. It is important to note that most choline in eggs is bound and not in free form, which changes how the nutrient is absorbed and digested. Miller et al., found that only 14% of choline from eggs is converted to TMAO, which is efficiently excreted and does not accumulate in the bloodstream [23].

Dietary supplements contributed minimal amounts of choline to the diet, since only small portions of the population consume choline-containing supplements. The House of Delegates of the American Medical Association (AMA) adopted Resolution 517 on June 13, 2017 in support evidence-based amounts of choline in all prenatal supplements for the purpose of ensuring that the baby’s 4brain and spinal cord develop properly [24]. Several forms of choline are available on the market, the most popular being choline chloride and choline bitartrate.

Educating individuals on foods that are rich in choline may assist in the public health effort. Particular public health emphasis should be given to pregnant and/or lactating women, since a large majority do not meet the AI and since choline is vital to the development of the fetus. Serum concentrations of choline in women are much less than that of breast milk [25]. Recommending supplementation and/or higher egg consumption (or a mixture of both for those concerned with dietary cholesterol intakes) in this subpopulation (and others) may be appropriate given that most adult women have lower than usual intakes of choline. Fortification of staple commodities and products may serve as an additional strategy to increase choline intakes across the population. These recommended strategies are not likely to increase choline intakes anywhere near the UL.

## 5. Conclusions

Many gender and life-stage subpopulations, as well as pregnant women in the U.S. do not currently meet the AI for choline. Only 8.03 ± 0.56% of adults and 8.51 ± 2.89 pregnant women meet the AI for choline. Children 2–3 years were the most likely to meet their gender and life-stage specific AI, followed by children 4–8 years. Adults 19+ years who consume eggs were more likely to meet their gender and life-stage AI as compared to non-consumers. Protein food (meat, poultry and seafood) consumption also increased usual choline intakes compared to non-consumers to a lesser degree, but did not result in substantial increases in the percent of individuals meeting the AI. No subpopulation exceeded the UL for choline. This research illustrates that it is extremely difficult to achieve the AI for choline without consuming eggs or taking a dietary supplement. Educating individuals on foods that are rich in choline, the fortification of staple food items, and/or encouragement of supplementation in certain subpopulations such as pregnant and/or lactating women may assist in public health efforts. Revision of the DRIs for choline to reflect the current peer-reviewed literature is critical.

## Figures and Tables

**Table 1:** Dietary reference intakes for choline.

Life-Stage Group	AI (mg/day)	UL (mg/day)
Infant		
0–6 Months	125	Not established
6–12 Months	150	Not established
Children		
1–3 Years	200	1000
4–8 Years	250	1000
9–13 Years	375	2000
Males		
14–18 Years	550	3000
≥19 Years	550	3500
Females		
14–18 Years	400	3000
≥19 Years	425	3500
Pregnancy		
All ages	450	Age-appropriate UL
Lactation		
All ages	550	Age-appropriate UL

AI = adequate intake; UL = tolerable upper intake level.

**Table 2:** Choline Intakes among individuals in the National Health and Nutrition Examination Survey (NHANES) 2009-2014 Datasets by Gender and Age ^a^.

Gender/Age	*N*	Usual Intake ^a^	Percentile						
			5 ^a^	25 ^a^	50 ^a^	75 ^a^	95 ^a^	% > AI	% > UL
**All**									
2–3 years	1316	224 ± 3.60	129 ± 3.95	176 ± 3.31	217 ± 3.58	264 ± 4.82	344 ± 8.41	60.7 ± 2.09	0.00 ± 0.00
4–8 years	2774	243 ± 2.45	141± 4.02	192 ± 2.79	235 ± 2.53	285 ± 3.49	369 ± 6.79	41.5 ± 1.50	0.00 ± 0.00
9–13 years	2559	257 ± 3.63	151 ± 4.88	205 ± 3.67	249 ± 3.66	301 ± 4.45	389 ± 7.78	6.63 ± 0.96	0.00 ± 0.00
14–18 years	2354	269 ± 4.96	159 ± 5.86	214 ± 5.08	262 ± 4.95	315 ± 5.47	405 ± 8.08	1.12 ± 0.29	0.00 ± 0.00
19–30 years	3288	330 ± 3.56	169 ± 3.76	240 ± 3.29	308 ± 3.75	399 ± 5.21	561 ± 9.75	6.59 ± 0.66	0.00 ± 0.00
31–50 years	5267	350 ± 2.97	184 ± 3.53	257 ± 3.05	327 ± 3.05	422 ± 4.58	591 ± 9.53	9.69 ± 0.77	0.00 ± 0.00
51–70 years	4975	342 ± 3.36	183 ± 3.72	254 ± 3.25	321 ± 3.60	409 ± 4.66	572 ± 8.91	8.42 ± 0.68	0.00 ± 0.00
71+ years	2244	307 ± 3.16	167 ± 3.61	232 ± 3.10	289 ± 3.45	364 ± 4.48	506 ± 7.77	4.39 ± 0.48	0.00 ± 0.00
**Male**									
2–3 years	658	246 ± 4.63	150 ± 4.11	199 ± 3.93	240 ± 4.48	287 ± 6.04	364 ± 9.39	74.3 ± 2.24	0.00 ± 0.00
4–8 years	1452	265 ± 3.11	162 ± 3.95	215 ± 2.98	258 ± 3.22	307 ± 4.35	390 ± 7.35	54.5 ± 1.85	0.00 ± 0.00
9–13 years	1279	283 ± 4.38	175 ± 4.94	230 ± 4.21	275 ± 4.33	328 ± 5.41	415 ± 8.45	11.2 ± 1.43	0.00 ± 0.00
14–18 years	1207	295 ± 5.18	184 ± 5.57	241 ± 5.16	288 ± 5.01	341 ± 5.96	431 ± 9.53	0.32 ± 0.13	0.00 ± 0.00
19–30 years	1702	392 ± 5.39	216 ± 4.92	305 ± 4.45	380 ± 5.16	466 ± 7.02	613 ± 12.18	10.5 ± 1.13	0.00 ± 0.00
31–50 years	2563	421 ± 4.99	236 ± 5.07	330 ± 4.13	408 ± 4.74	498 ± 6.63	652 ± 11.57	15.3 ± 1.25	0.00 ± 0.00
51–70 years	2455	408 ± 5.15	227 ± 5.33	319 ± 4.70	396 ± 4.98	483 ± 6.58	633 ± 11.07	13.0 ± 1.11	0.00 ± 0.00
71+ years	1099	363 ± 4.80	197 ± 5.87	280 ± 4.86	351 ± 4.97	432 ± 5.93	571 ± 9.55	6.59 ± 0.74	0.00 ± 0.00
**Female**									
2–3 years	658	201 ± 3.64	118 ± 4.37	160 ± 3.47	195 ± 3.53	235 ± 4.75	303 ± 7.74	46.7 ± 2.56	0.00 ± 0.00
4–8 years	1322	218 ± 2.72	130 ± 4.28	174 ± 3.50	211 ± 2.84	255 ± 3.43	329 ± 6.99	27.4 ± 1.67	0.00 ± 0.00
9–13 years	1280	233 ± 3.75	140 ± 4.74	187 ± 4.22	226 ± 3.88	272 ± 4.31	347 ± 6.87	2.46 ± 0.51	0.00 ± 0.00
14–18 years	1147	244 ± 4.80	147 ± 5.79	197 ± 4.94	237 ± 4.74	283 ± 5.27	361 ± 7.93	1.90 ± 0.49	0.00 ± 0.00
19–30 years	1586	257 ± 4.14	152 ± 4.38	206 ± 3.92	250 ± 4.09	301 ± 5.16	386 ± 8.13	2.08 ± 0.49	0.00 ± 0.00
31–50 years	2704	279 ± 3.32	168 ± 4.27	224 ± 3.51	271 ± 3.33	325 ± 4.23	415 ± 7.39	4.06 ± 0.68	0.00 ± 0.00
51–70 years	2520	280 ± 3.88	169 ± 4.17	226 ± 3.60	273 ± 3.77	327 ± 4.98	416 ± 8.29	4.16 ± 0.79	0.00 ± 0.00
71+ years	1145	266 ± 4.02	158 ± 4.35	213 ± 4.05	259 ± 4.03	311 ± 4.83	397 ± 8.04	2.76 ± 0.58	0.00 ± 0.00
**Pregnant ^b^**									
13–44 years	593	319 ± 9.89	187 ± 13.33	254 ± 10.63	309 ± 9.75	373 ± 13.22	490 ± 27.85	8.51 ± 2.89	0.00 ± 0.00

AI = Adequate intake; UL = Tolerable upper intake level; ^a^ Data are presented as mg/day ± standard error; ^b^ Pregnant women were enrolled in the NHANES 2005–2014 datasets.

**Table 3:** Choline intakes among consumers vs. non-consumers of eggs ^a,b,c^.

Gender/Age	Day ^d^	Non-Consumers			Consumers			*p*-Value ^e^
		*N*	Usual Intake ^a^	% > AI	*N*	Usual Intake ^a^	% > AI	
**All**								
19–30 years	1	2653	286 ± 3.54	1.75 ± 0.33	635	534 ± 9.27	58.1 ± 2.78	<0.0001
	2	2291	279 ± 3.63	1.22 ± 0.26	997	454 ± 6.69	24.2 ± 3.51	<0.0001
31–50 years	1	4160	305 ± 2.85	3.18 ± 0.40	1107	545 ± 8.61	64.1 ± 2.66	<0.0001
	2	3504	301 ± 3.08	2.49 ± 0.36	1763	453 ± 6.24	23.0 ± 2.90	<0.0001
51–70 years	1	3762	297 ± 3.39	2.54 ± 0.37	1213	520 ± 8.48	57.4 ± 2.91	<0.0001
	2	2069	290 ± 3.96	1.86 ± 0.36	1906	429 ± 5.44	13.7 ± 2.45	<0.0001
71+ years	1	1723	266 ± 3.19	1.01 ± 0.20	521	463 ± 8.54	37.3 ± 2.71	<0.0001
	2	1410	265 ± 3.43	0.79 ± 0.20	834	375 ± 6.19	3.19 ± 1.50	<0.0001
**Male**								
19–30 years	1	1347	339 ± 5.47	3.08 ± 0.61	355	610 ± 15.9	65.3 ± 3.87	<0.0001
	2	1163	327 ± 5.16	1.99 ± 0.43	539	539 ± 11.4	42.4 ± 6.20	<0.0001
31–50 years	1	1988	366 ± 4.98	5.53 ± 0.74	575	637 ± 14.0	72.1 ± 3.36	<0.0001
	2	1677	360 ± 5.41	4.38 ± 0.69	886	543 ± 10.7	44.5 ± 5.48	<0.0001
51–70 years	1	1812	354 ± 5.43	4.40 ± 0.62	643	599 ± 13.7	62.0 ± 4.29	<0.0001
	2	1477	346 ± 6.17	3.28 ± 0.67	978	509 ± 9.84	28.1 ± 5.04	<0.0001
71+ years	1	801	312 ± 4.66	1.64 ± 0.31	298	522 ± 10.3	38.0 ± 3.44	<0.0001
	2	668	309 ± 4.46	1.64 ± 0.31	431	447 ± 8.00	7.44 ± 3.36	<0.0001
**Female**								
19–30 years	1	1306	225 ± 3.77	0.36 ± 0.14	280	427 ± 11.4	48.5 ± 5.44	<0.0001
	2	1128	224 ± 4.14	0.31 ± 0.13	458	341 ± 7.62	0.00 ± 0.03	<0.0001
31–50 years	1	2172	246 ± 2.85	0.85 ± 0.26	532	438 ± 8.63	54.1 ± 4.18	<0.0001
	2	1827	243 ± 3.46	0.68 ± 0.29	877	358 ± 6.19	0.00 ± 0.31	<0.0001
51–70 years	1	1950	246 ± 3.03	0.83 ± 0.27	570	435 ± 8.66	52.6 ± 4.16	<0.0001
	2	1592	239 ± 3.38	0.64 ± 0.25	928	352 ± 6.35	0.00 ± 0.10	<0.0001
71+ years	1	922	234 ± 4.01	0.56 ± 0.19	223	403 ± 8.04	37.0 ± 3.79	<0.0001
	2	742	234 ± 4.62	0.53 ± 0.23	403	316 ± 7.63	0.00 ± 0.00	<0.0001

AI = Adequate intake; UL = Tolerable upper intake level; ^a^ Data are presented as mg/d ± standard error; ^b^ No incidence of any population exceeding the UL for choline; ^c^ Non-consumers are those who did not report consuming whole eggs (i.e., they could have consumed products containing eggs); ^d^ Day 1 denotes consumer on day one. Day 2 denotes consumer on either NHANES intake day; ^e^ Comparison of usual intake of non-consumers vs. consumers.

**Table 4:** Food pattern modeling incorporating eggs as a substitute for red and/or processed meat.

Nutrient	Healthy US-Style 2000 Kcal Pattern (%DV)	Eggs (1 oz eq.)	1 Egg/Day	2 Eggs/Day
Energy (kcal)	2003	78	2019	2048
Protein (g)	91 (18%) ^1^	6.30	90.5	89.7
Total CHO (g)	256 (51%) ^1^	0.60	256	256
Fiber (g)	31 (111%)	0.00	31.0	31.0
Total fat (g)	72 (32%) ^1^	5.30	73.9	77.2
Sat fat (g)	18.7 (8%) ^1^	1.63	19.2	20.1
MUFA (g)	26.2 (12%) ^1^	2.04	26.9	28.0
PUFA (g)	22.5 (10%) ^1^	0.71	22.8	23.4
Cholesterol (mg)	215 (72%)	187	310	477
Calcium (mg)	1274 (98%)	25.0	1287	1310
Iron (mg)	17 (94%)	0.60	17.1	17.2
Magnesium (mg)	352 (84%)	5.00	351	350
Phosphorus (mg)	1717 (137%)	86.0	1729	1750
Potassium (mg)	3348 (71%)	63.0	3331	3301
Sodium (mg)	1787 (78%)	62.0	1750	1685
Zinc (mg)	14 (127%)	0.50	13.6	12.9
Copper (mg)	1.4 (156%)	0.00	1.40	1.40
Manganese (mg)	4 (174%)	0.01	4.00	4.00
Selenium (mg)	110 (200%)	15.4	114	121
Vitamin A (RAE)	898 (100%)	74.5	934	997
Vitamin E (mg AT)	10.2 (68%)	0.52	10.5	10.9
Vitamin D (IU)	274 (69%)	43.5	296	336
Vitamin C (mg)	117 (130%)	0.00	117	117
Thiamin (mg)	1.7 (142%)	0.03	1.70	1.70
Riboflavin (mg)	2.1 (162%)	0.26	2.20	2.40
Niacin (mg)	24 (150%)	0.03	23.1	21.4
Vitamin B6 (mg)	2.3 (135%)	0.06	2.30	2.20
Vitamin B12 (mcg)	6.8 (283%)	0.56	6.80	6.70
Choline (mg)	349 (63%)	147	418	539
Vitamin K (mcg)	139 (116%)	0.00	139	139

**Table 5:** Choline intakes among consumers vs. non-consumers of protein foods (meat, poultry and seafood).

Gender/Age	Day ^c^	Non-Consumers ^b^			Consumers ^b^			*P*-value ^d^
		N	Usual Intake ^a^	% > AI	N	Usual Intake ^a^	% > AI	
**All**								
19–30 years	1	196	232 ± 19.0	2.35 ± 1.53	3092	336 ± 3.89	6.76 ± 0.81	<0.0001
	2	84	227 ± 23.7	3.15 ± 2.13	3204	332 ± 3.64	6.27 ± 0.72	<0.0001
31–50 years	1	332	230 ± 12.4	1.76 ± 0.92	4935	359 ± 3.36	10.6 ± 0.89	<0.0001
	2	149	251 ± 20.0	5.08 ± 2.12	5118	353 ± 3.01	9.66 ± 0.75	<0.0001
51–70 years	1	317	248 ± 11.4	2.92 ± 1.12	4658	349 ± 3.04	8.79 ± 0.71	<0.0001
	2	118	235 ± 20.5	3.67 ± 1.71	4857	344 ± 3.12	8.31 ± 0.64	<0.0001
71+ years	1	174	221 ± 10.2	1.58 ± 0.75	2070	314 ± 3.53	4.58 ± 0.58	<0.0001
	2	64	247 ± 23.9	5.74 ± 2.62	2180	309 ± 3.37	4.13 ± 0.49	<0.0001
**Male**								
19–30 years	1	88	285 ± 34.9	4.69 ± 3.42	1614	398 ± 6.42	10.5 ± 1.33	<0.0001
	2	39	255 ± 39.3	5.86 ± 3.67	1663	394 ± 5.63	10.1 ± 1.19	<0.0001
31–50 years	1	135	284 ± 21.1	4.49 ± 2.24	2428	429 ± 5.42	16.1 ± 1.42	<0.0001
	2	67	289 ± 27.0	8.77 ± 3.37	2496	425 ± 5.26	15.4 ± 1.33	<0.0001
51–70 years	1	136	319 ± 28.0	7.92 ± 3.24	2319	413 ± 4.99	13.1 ± 1.23	<0.0001
	2	52	270 ± 48.8	6.86 ± 4.03	2403	410 ± 4.62	12.6 ± 1.14	<0.0001
71+ years	1	62	284 ± 23.2	4.46 ± 2.34	1037	367 ± 5.10	6.33 ± 0.87	<0.0001
	2	28	347 ± 52.1	14.1 ± 6.99	1071	363 ± 4.97	5.96 ± 0.81	0.0004
**Female**								
19–30 years	1	108	180 ± 12.6	0.07 ± 0.21	1478	264 ± 4.48	2.58 ± 0.58	<0.0001
	2	45	202 ± 24.7	0.95 ± 1.66	1541	258 ± 4.16	1.99 ± 0.49	<0.0001
31–50 years	1	197	198 ± 11.4	0.31 ± 0.38	2507	287 ± 3.52	4.84 ± 0.82	<0.0001
	2	82	215 ± 20.8	1.90 ± 1.58	2622	281 ± 3.32	3.99 ± 0.65	<0.0001
51–70 years	1	181	209 ± 12.6	0.36 ± 0.65	2339	287 ± 3.65	4.98 ± 0.80	<0.0001
	2	66	214 ± 15.6	1.92 ± 1.36	2454	282 ± 3.82	4.25 ± 0.75	<0.0001
71+ years	1	112	193 ± 9.15	0.23 ± 0.27	1033	272 ± 3.74	3.23 ± 0.63	<0.0001
	2	36	190 ± 16.4	0.73 ± 0.70	1109	267 ± 3.77	2.66 ± 0.52	<0.0001

AI = Adequate intake; UL = Tolerable upper intake level; ^a^ Data are presented as mg/d ± standard error; ^b^ No incidence of any population exceeding the UL for choline; ^c^ Day 1 denotes consumer on day one. Day 2 denotes consumer on either NHANES intake day; ^d^ Comparison of usual intake of non-consumed.

**Table 6:** Contribution of dietary supplements by frequency to total usual choline intakes.

Gender/Age	Consumers ^b^		1–10 d/mo ^b^		11–20 d/mo ^b^		21–30 d/mo ^b^	
	*N*	Usual Intake ^a^	*N*	Usual Intake ^a^	*N*	Usual Intake ^a^	*N*	Usual Intake
**All**								
2–3 years	268	12.6 ± 1.22	34	4.00 ± 1.23	75	8.97 ± 1.45	159	16.4 ± 1.73
4–8 years	555	13.9 ± 1.39	82	3.45 ± 0.80	134	12.7 ± 1.79	339	16.7 ± 1.73
9–13 years	333	15.1 ± 1.46	69	2.81 ± 0.84	68	10.9 ± 1.40	196	20.4 ± 1.89
14–18 years	130	12.2 ± 2.03	22	1.38 ± 0.63	36	12.1 ± 2.78	72	16.3 ± 3.16
19–30 years	169	21.2 ± 4.67	42	4.07 ± 0.63	35	8.31 ± 2.45	92	31.3 ± 8.32
31–50 years	310	14.1 ± 2.01	67	4.54 ± 1.17	77	10.3 ± 1.88	166	19.0 ± 3.54
51–70 years	200	39.3 ± 6.10	22	2.53 ± 0.57	22	6.96 ± 2.03	156	47.4 ± 7.55
71+ years	76	42.2 ± 12.33	7	1.94 ± 1.04	5	45.3 ± 25.46	64	47.5 ± 14.66
**Male**								
2–3 years	139	12.1 ± 1.52	22	4.25 ± 1.86	43	8.74 ± 1.45	74	17.6 ± 2.78
4–8 years	296	14.1 ± 1.70	43	3.78 ± 0.96	66	13.1 ± 2.02	187	16.6 ± 2.20
9–13 years	171	14.3 ± 2.08	35	3.35 ± 1.47	36	6.92 ± 2.24	100	20.3 ± 2.56
14–18 years	70	12.5 ± 2.83	11	1.46 ± 0.80	21	13.2 ± 4.23	38	15.2 ± 4.05
19–30 years	87	29.1 ± 8.73	21	4.25 ± 1.00	16	9.62 ± 4.24	50	42.3 ± 14.1
31–50 years	131	14.0 ± 2.31	33	4.41 ± 1.17	26	12.6 ± 4.05	72	18.9 ± 4.51
51–70 years	73	44.0 ± 8.80	7	1.84 ± 0.35	13	8.33 ± 2.89	53	57.9 ± 13.2
71+ years	35	47.0 ± 15.19	4	1.37 ± 0.86	1	100 ± 0.00	30	54.1 ± 19.4
**Female**								
2–3 years	129	13.0 ± 1.71	12	3.56 ± 0.81	32	9.34 ± 2.55	85	15.7 ± 2.32
4–8 years	259	13.7 ± 1.95	39	2.94 ± 1.03	68	12.2 ± 2.41	152	16.9 ± 2.50
9–13 years	162	16.1 ± 1.59	34	2.19 ± 0.69	32	14.9 ± 1.83	96	20.5 ± 2.25
14–18 years	60	11.8 ± 2.98	11	1.30 ± 0.89	15	10.8 ± 3.34	34	18.5 ± 4.46
19–30 years	82	12.9 ± 2.59	21	3.83 ± 0.63	19	7.62 ± 3.06	42	18.3 ± 4.51
31–50 years	179	14.2 ± 2.61	34	4.74 ± 2.42	51	8.87 ± 2.00	94	19.1 ± 4.27
51–70 years	127	36.8 ± 7.66	15	3.11 ± 1.03	9	5.48 ± 2.56	103	42.5 ± 8.59
71+ years	41	38.6 ± 14.25	3	2.97 ± 2.15	4	8.48 ± 4.65	34	43.0 ± 16.2
**Pregnant**								
13–44 years	30	14.4 ± 3.18	1	9.17 ± 0.00	6	7.44 ± 8.15	23	15.4 ± 3.62

^a^ Data are presented as mg/d ± standard error; ^b^ No incidence of any population exceeding the UL for choline.
